# Chemo-sensors development based on low-dimensional codoped Mn_2_O_3_-ZnO nanoparticles using flat-silver electrodes

**DOI:** 10.1186/1752-153X-7-60

**Published:** 2013-03-28

**Authors:** Mohammed M Rahman, George Gruner, Mohammed Saad Al-Ghamdi, Muhammed A Daous, Sher Bahadar Khan, Abdullah M Asiri

**Affiliations:** 1 Center of Excellence for Advanced Materials Research (CEAMR), King Abdulaziz University, P.O. Box 80203, Jeddah, 21589, Saudi Arabia; 2 Chemistry Department, Faculty of Science, King Abdulaziz University, P.O. Box 80203, Jeddah, 21589, Saudi Arabia; 3 Department of Physics, University of California Los Angeles, 405 Hilgard Avenue, Los Angeles, California, 90095, USA; 4 Physics Department, Faculty of Science, King Abdulaziz University, P.O. Box 80203, Jeddah, 21589, Saudi Arabia; 5 Chemical Engineering, Faculty of Engineering, King Abdulaziz University, Jeddah, 21589, Saudi Arabia

**Keywords:** Doped Mn_2_O_3_-ZnO nanoparticles, Wet-chemical method, Powder X-ray diffraction, 4-nitrophenol, I-V technique, X-ray photoelectron spectroscopy, Sensitivity

## Abstract

**Background:**

Semiconductor doped nanostructure materials have attained considerable attention owing to their electronic, opto-electronic, para-magnetic, photo-catalysis, electro-chemical, mechanical behaviors and their potential applications in different research areas. Doped nanomaterials might be a promising owing to their high-specific surface-area, low-resistances, high-catalytic activity, attractive electro-chemical and optical properties. Nanomaterials are also scientifically significant transition metal-doped nanostructure materials owing to their extraordinary mechanical, optical, electrical, electronic, thermal, and magnetic characteristics. Recently, it has gained significant interest in manganese oxide doped-semiconductor materials in order to develop their physico-chemical behaviors and extend their efficient applications. It has not only investigated the basic of magnetism, but also has huge potential in scientific features such as magnetic materials, bio- & chemi-sensors, photo-catalysts, and absorbent nanomaterials.

**Results:**

The chemical sensor also displays the higher-sensitivity, reproducibility, long-term stability, and enhanced electrochemical responses. The calibration plot is linear (r^2^ = 0.977) over the 0.1 nM to 50.0 μM 4-nitrophenol concentration ranges. The sensitivity and detection limit is ~4.6667 μA cm^-2^ μM^-1^ and ~0.83 ± 0.2 nM (at a Signal-to-Noise-Ratio, SNR of 3) respectively. To best of our knowledge, this is the first report for detection of 4-nitrophenol chemical with doped Mn_2_O_3_-ZnO NPs using easy and reliable I-V technique in short response time.

**Conclusions:**

As for the doped nanostructures, NPs are introduced a route to a new generation of toxic chemo-sensors, but a premeditate effort has to be applied for doped Mn_2_O_3_-ZnO NPs to be taken comprehensively for large-scale applications, and to achieve higher-potential density with accessible to individual chemo-sensors. In this report, it is also discussed the prospective utilization of Mn_2_O_3_-ZnO NPs on the basis of carcinogenic chemical sensing, which could also be applied for the detection of hazardous chemicals in ecological, environmental, and health care fields.

## Introduction

Semiconductor codoped nanomaterials have received significant interest due to their electronic, optoelectronic, magnetic, catalytical, electro-chemical, mechanical behaviors and their potential applications in different research areas. Semiconductor nanomaterials might be a promising due to their high-specific surface-area, low-resistances, high-catalytic activity, attractive electrochemical and optical properties [1,2]. Nanomaterials are also scientifically important codoped nanostructure materials owing to their extraordinary mechanical, optical, electrical, electronic, thermal, and magnetic characteristics. Lately, it has attained significant attention in manganese doped-semiconductor materials in order to develop their physic-chemical behaviors and extend their efficient applications [3-5]. It has not only investigated the basic of magnetism, but also has huge potential in scientific features such as magnetic materials, bio & chemi-sensors, photo-catalysts, and absorbent nanomaterials [6-9]. Recently, very few articles are published based on transition-metal doped semiconductor nanomaterials synthesis and investigated the magnetic behaviors and potential applications only [10-13]. Here, it is prepared codoped Mn_2_O_3_-ZnO NPs by easy, facile, economical, non-toxic, repeatable, and reliable low-temperature wet-chemical technique. The nanostructure and morphology of the codoped Mn_2_O_3_-ZnO NPs were examined and potentially applied for the enhancement of higher-sensitive 4-nitrophenol chemo-sensor at room condition. Generally, chemo-sensing exploration have been developed with the transition-metal oxides nanostructures for the recognition and quantification of various toxic-chemicals such as phenyl-hydrazine, methanol, formaldehyde, ethanol, chloroform, dichloromethane etc., which are not ecologically safe and friendly [14-18]. The sensing mechanism with doped semiconductor metal oxides thin-film used primarily the properties of meso-porous thin-film generated by the physi-sorption and chemisorptions methods. The hazardous chemical detection is depended on the current responses of the fabricated thin-film, which cause by the presence of chemical components in the reaction-format in aqueous phase [19-21]. The key efforts are based on recongnition the least amount of 4-nitrophenol necessary for the fabricated Mn_2_O_3_-ZnO NPs chemo-sensors for electrochemical investigation.

Phenolic compounds have attained significant interest in last decade owing to their eco-toxic effects on human health, ecological, and environmental fields. These toxic compounds (i.e., 4-nitrophenol) are prepared using a number of polluting techniques, such as industry-related ways of plastic, pesticides, paint, drugs, composites, antioxidant, petroleum, and paper production [22]. The 4-nitrophenol is recognized for its hazardous nature, carcinogenetic, toxicity, and persistence in the environment, which is become a common pollutant in nature and waste water [23]. Because of its high solubility and stability in water, it has been also found in freshwater, sea environments and has been detected in industrial wastewaters and is difficult to degrade by conventional method. It is concerned in most of the degradation pathways of organo-phosphorous pesticides, which are decomposed in soil and water to form 4-nitrophenol as an intermediate or final-product in the reaction systems [24,25]. Therefore, 4-nitrophenol is integrated in the Environmental Protection Agency List of Priority Pollutants (EPALPP) [26]. Therefore, it is straight away desirable to fabricate a chemo-sensor for the detection of organic pollutants to accumulate the environment and human health. There is focused a significant attention for the development of simple, reliable, and ultra-sensitive in various detection methodology based on codoped nanomaterials. Generally, the detection of toxic 4-nitrophenol is consummated using chromatographic techniques, such as gas-chromatography [27,28], high-performance liquid chromatography [29,30], liquid chromatography connected with mass-spectroscopy [31], and capillary-electrophoresis [32]. Electrochemical technique, which can offer fast, reliable, and direct real-time monitoring is one of the most utilized methods in the determination of nitro-phenolic stuffs. Electro-analytical techniques have been performed for 4-nitrophenol detection and quantification with a modified glassy carbon electrode [33,34] hanging mercury drop electrode [35] and boron-doped diamond electrode [36]. The analytical signal is derived from the four-electron reduction of the nitro-group [37] or by the direct two-electron oxidation of phenol to the corresponding o-benzoquinone [38-40]. Electrochemical chemo-sensors have attained huge interest in the recognition and quantification of environmental unsafe chemicals due to their reliable and fast response and determination [41-44]. Chemo-sensor technology plays a significant task in ecological protection that usually caused by environmental contamination and unintended seepage of harmful chemicals, which is a huge-menace for eco-systems. Thus for the attention of ecological and health monitoring, it is important to fabricate easy, simple, reproducible, reliable, and inexpensive chemo-sensors to detect toxic chemicals in aqueous systems. The sensitivity and low-detective of electrochemical chemo-sensor energetically dependent on the size, structure and properties of fabricated electrode doped nanomaterials. Hence doped nanostructure materials have received much attention and have widely been used as a redox mediator in chemo-sensors [45-48].

Codoped nanomaterial is largely established for the recognition of toxic chemicals in electro-chemical control method owing to their numerous benefits over conventional chemical methods in term of large-surface area for examining in medical, health-care and environmental fields [49-56]. In general electro-analytical technique, it was executed the slower responses, surface-fouling, noises, flexible-responses, and smaller dynamic-range and lower-sensitivity with bared codoped nanomaterials surfaces for chemical recognition. Therefore, the modification of the chemo-sensor surface with doped metal oxides nanostructure materials is urgently required to achieve higher sensitive, repeatability, and stable responses. Therefore, an easy and reliable I-V electrochemical approach is immediately needed for relatively simple, appropriate, and economical instrumentation which displays higher-sensitivity and lower-detection limits compared to general techniques. Here, a consistent, large-scale, and highly responsive I-V method is applied for detection of 4-nitrophenol chemical by codoped Mn_2_O_3_-ZnO NPs. The present approach represents a consistent, sensitive, low-sample volume, ease to handle, and specific electrochemical methods over the existing UV, CV, LC-MS, LSV, FL, and HPLC methods [57-60]. The simple coating technique for preparation of nanomaterials thin-film with conducting coating agents is developed for the fabrication of doped Mn_2_O_3_-ZnO NPs films. Here, low-dimensional doped Mn_2_O_3_-ZnO NPs films with conducting coating agents are synthesized and detected 4-nitrophenol in phosphate buffer solution (PBS) phase by reliable I-V method. To best of our knowledge, this is the first report for detection of 4-nitrophenol chemical with doped Mn_2_O_3_-ZnO NPs using easy and reliable I-V technique in short response time.

## Experimental sections

### Materials and methods

Manganese chloride (MnCl_2_.4H_2_O), zinc chloride (ZnCl_2_), 4-nitrophenol, ammonium hydroxide (25%), Ethyl cellulose (EC), Disodium phosphate, Butyl carbitol acetate (BCA), Ethanol, Monosodium phosphate, and all chemicals utilized were of analytical grade and obtained from Sigma-Aldrich Company. Stock solution of 1.0 M 4-nitrophenol was synthesized in double distilled water. The doped Mn_2_O_3_-ZnO NPs was investigated with UV/visible spectroscopy (Lamda-950, Perkin Elmer, Germany). FT-IR spectra were recorded for Mn_2_O_3_-ZnO NPs with a spectrophotometer (Spectrum-100 FT-IR) in the mid-IR range, which was acquired from Perkin Elmer, Germany. Raman station 400 (Perkin Elmer, Germany) was exploited to investigate the Raman shift of Mn_2_O_3_-ZnO NPs using radiation source (Ar^+^ laser line, λ: ~513.4 nm). The XPS measurements were executed on a Thermo Scientific K-Alpha KA1066 spectrometer (Germany). Monochromatic AlKα x-ray radiation sources were used as excitation sources, where beam-spot size was kept in 300.0 μm. The spectrum was recorded in the fixed analyzer of transmission mode, where pass-energy was kept at 200.0 eV. The scanning of the spectra was performed at lower pressures (<10^−8^ Torr). The X-ray powder (XRD) diffraction prototypes were measured with X-ray diffractometer (XRD; X’Pert Explorer, PANalytical diffractometer) prepared with Cu-Kα1 radiation (*λ* = 1.5406 nm) by a generator voltage (~40.0 kV) and current (~35.0 mA) applied for the measurement. Morphology of codoped Mn_2_O_3_-ZnO NPs was evaluated on FE-SEM instrument (FESEM; JSM-7600 F, Japan). Elemental analysis (EDS) was investigated for doped Mn_2_O_3_-ZnO NPs using from JEOL, Japan. I-V technique was used for sensing NPs modified sensor electrode by Electrometer (Kethley, 6517A, Electrometer, USA) at room conditions.

### Synthesis and growth mechanism of codoped Mn_2_O_3_-ZnO NPs

Initially manganese chloride (MnCl_2_.4H_2_O) and zinc chloride (ZnCl_2_) were gradually dissolved into the de-ionized water to prepare 0.1 M concentration separately at room temperature. After addition of NH_4_OH into the mixture of metal chloride solution, it was stirred slowly for several minutes at room condition. Mn_2_O_3_-ZnO NPs have been synthesized by adding equi-molar concentration of manganese chloride and zinc chloride as starting (reducing) materials into reaction-cell (in Teflon-line auto-clave) for 12 hours. Then the solution pH is attuned (at 10.5) by using prepared NH_4_OH and put into the auto-clave cell. The starting materials of MnCl_2_ and ZnCl_2_ were employed without further purification for co-precipitation method to codoped Mn_2_O_3_-ZnO nanoparticles composition. Again reducing agent (NH_4_OH) was added drop-wise into the vigorously stirred MnCl_2_ and ZnCl_2_ solutions mixture to produce a significant doped precipitate.

The growth mechanism of doped Mn_2_O_3_-ZnO NPs can be explained based on chemical reactions and nucleation, and growth of doped Mn_2_O_3_-ZnO crystals. The probable reaction mechanisms are anticipated for achieving the codoped Mn_2_O_3_-ZnO nanomaterials, which are appended in below.

(1) $$\mathrm{ZnC}1_{2\left(s\right)}\to Zn_{\mathrm{aq}}^{2+}+2C1_{\mathrm{aq}}^{-}$$

(2) $$\mathrm{MnC}1_{2\left(s\right)}\to Mn_{\left(\mathrm{aq}\right)}^{2+}+2c1_{\left(\mathrm{aq}\right)}^{-}$$

(3) $$NH_{4}OH_{\left(\mathrm{aq}\right)}\to NH_{4}^{+}+OH^{-}$$

(4) $$4Mn_{\left(\mathrm{aq}\right)}^{2+}+2\mathrm{Zn}H_{\left(\mathrm{aq}\right)}^{2+}+80H_{\left(\mathrm{aq}\right)}^{-}\to 2Mn_{2}O_{3}-\mathrm{Zn}O_{\left(s\right)}+4H_{2}O$$

The precursors of MnCl_2_ and ZnCl_2_ are soluble in alkaline medium (NH_4_OH reagent) according to the equation of (i) - (iii). After addition of NH_4_OH into the mixture of metal chlorides solution, it was strongly stirred for several minutes at room temperature. The reaction is development gradually according to the equation (iv). Then the resultant solution was washed systematically with ethanol, acetone and kept for drying at room temperature. During the total preparative procedure, NH_4_OH acts a pH buffer to control the pH value of the solution and slow donate of OH^–^ ions. When the concentrations of the Mn^2+^, Zn^2+^, and OH^-^ ions are reached above the critical value, the precipitation of doped Mn_2_O_3_-ZnO nuclei begin to start. As there is higher concentration of Zn^2+^ ion in the solution, the nucleation of doped Mn_2_O_3_-ZnO crystals become easier due to the lower-activation energy barrier of heterogeneous nucleation. However, as the concentration of Zn^2+^ subsistence, a number of larger doped Mn_2_O_3_-ZnO crystals with a spherical particle-shape morphology form in nano-level. The shape of codoped Mn_2_O_3_-ZnO NPs is approximately consistent with the growth pattern of codoped Mn_2_O_3_-ZnO crystals [61,62]. Finally, the as-grown codoped Mn_2_O_3_-ZnO NPs products were calcined at 400.0°C for 4 hours in the furnace (Barnstead Thermolyne, 6000 Furnace, USA). The calcined doped nanomaterials were synthesized in detail in terms of their morphological, structural, optical properties, and applied for 4-nitrophenol chemical sensing.

#### Fabrication of AgE using doped Mn_2_O_3_-ZnO NPs

**Figure 1:**
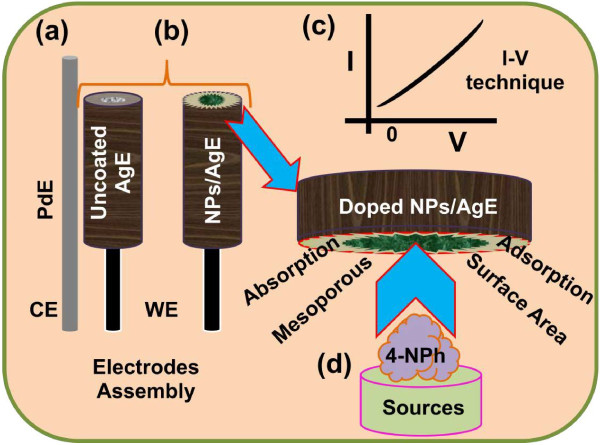
**Fabrication process and detection mechanism of 4-nitrophenol using codoped Mn**_**2**_**O**_**3**_**-ZnO NPs coated AgE.** (**a**) PdE used as counter electrode, (**b**) fabrication of uncoated and coated working electrode, AgE by NPs, (**c**) expected outcome by I-V technique, and (**d**) detection mechanism of 4-nitrophenol by doped Mn_2_O_3_-ZnO NPs/AgE chemo-sensor.

Phosphate buffer solution (PBS, 0.1 M, pH 7.0) is arranged by properly mixing Na_2_HPO_4_ (0.2 M) and NaH_2_PO_4_ (0.2 M) solution in 100.0 mL de-ionize water. Flat AgE is fabricated by doped Mn_2_O_3_-ZnO NPs with butyl carbitol acetate (BCA) and ethyl cellulose (EC) as conducting coating agents. Subsequently, the fabricated electrodes are transferred into the oven at 65.0°C for 12 hours until the film is totally dry, consistent, and stable. An electro-chemical cell is prepared with codoped Mn_2_O_3_-ZnO NPs coated silver electrode as a working electrode and palladium wire is employed as a counter electrodes. 4-nitrophenol (~1.0 M) is diluted at different concentration in DI water and used as a target chemical. Amount of 0.1 M PBS is kept constant in the small-beaker as 10.0 mL during the chemical analysis. Analyte solution is made with various concentration of 4-nitrophenol from 1.0 nM to 1.0 M. The sensitivity is calculated from the current-slope vs. analyte concentration from the calibration stature by considering the active surface area of doped Mn_2_O_3_-ZnO NPs fabricated chemo-sensors. Electrometer is properly utilized as a voltage sources for reliable I-V method in two electrodes assembly. With high-mechanical strength, good-conductivity, highly stability, large-surface area, and extremely miniaturized dimension of codoped Mn_2_O_3_-ZnO NPs have been extensively used in chemo-sensor modification and fabrication of 4-nitrophenol detection. The codoped Mn_2_O_3_-ZnO NPs were applied for the detection of 4-nitrophenol in liquid-phase system at room conditions. Initially, the NPs thin-film was prepared using conducting binders (EC and BCA) and embedded on the flat AgE electrode. The development and fabrication techniques are exhibited in the schematic diagram (Figure 1). The PdE and doped Mn_2_O_3_-ZnO NPs fabricated AgE is used as counter and working electrodes respectively, which is presented in Figure 1a-b. The 4-nitrophenol was employed as a target analytes in PBS buffer phase. The proposed electrical signals in presence of 4-nitrophenol chemical have been presented using I-V technique according to the Figure 1c. The physi-sorption etiquettes (absorption/adsorption) and detection method of doped Mn_2_O_3_-ZnO NPs are presented in the Figure 1d. Here the target analyte chemicals are adsorbed/absorbed onto the developed doped Mn_2_O_3_-ZnO NPs surfaces in huge amount owing to the meso-porous natures and large-active surface area of NPs in buffer phase respectively.

## Results and discussions

### Optical properties

**Figure 2:**
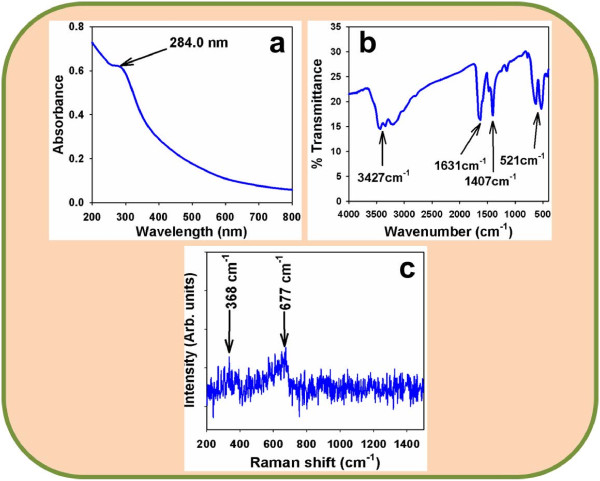
**Investigation of optical properties for doped Mn2O3-ZnO NPs at room conditions.** (**a**) UV/visible (range, 200.0 ~ 800.0 nm), (**b**) FT-IR spectroscopy (range, 450.0 ~ 4000.0 cm-1), and (**c**) Raman spectrum (200.0 ~ 1450.0 cm-1).

The optical behavior of the codoped Mn_2_O_3_-ZnO NPs is one of the important features for the evaluation of its photo-catalytic property. The optical absorption spectra of NPs are investigated by UV-visible spectrophotometer in the visible range (200.0 to 800.0 nm). From the absorption spectra, it has been investigated the absorbance of the doped Mn_2_O_3_-ZnO NPs is about ~284.0 nm, which is presented in Figure 2a. Band gap energy (E_bg_) is executed based on the most absorption band of NPs and originated to be ~4.50704 eV, according to following formula (v).

(5) $$E_{\mathrm{bg}}=\frac{1240}{\lambda }\left(\mathrm{eV}\right)$$

Where E_bg_ is the band-gap energy and λ_max_ is the wavelength (~284.0 nm) of the doped Mn_2_O_3_-ZnO NPs. No extra peak related with contaminants and structural defects were found in the spectrums, which confirmed that the prepared NPs control crystallinity of codoped Mn_2_O_3_-ZnO NPs [63,64].

The codoped Mn_2_O_3_-ZnO NPs are also investigated from the atomic and molecular vibrations. To investigate the vibration of materials, FT-IR spectrum mostly in the area of 450.0-4000.0 cm^-1^ is measured. Figure 2b displays the FT-IR spectrum of the Mn_2_O_3_-ZnO NPs. It represents band at 521.0, 1407.0, 1631.0, and 3427.0 cm^-1^. These executed wide vibration band (at 521.0 cm^-1^) could be assigned as metal-oxygen (Mn-O and Zn-O modes) stretching vibrations [65,66], which verified the pattern of doped Mn_2_O_3_-ZnO NPs. The additional experimental vibration bands may be allocated to O-H stretching (3427 cm^−1^), C-O stretching vibration (1631.0 cm^−1^), and O-H bending vibration (1407 cm^−1^). The absorption bands at 1407, 1631 and 3427 cm^-1^ usually displays from water and carbon dioxide, which generally semiconductor nanostructure materials absorbed from the surroundings owing to their meso-porous nature. Finally, the resultant vibration bands at lower-frequencies areas recommended the formation of codoped Mn_2_O_3_-ZnO NPs.

Raman spectroscopy is a spectroscopic method employs to reveal vibrational, rotational and other low-frequency phases in Raman active compounds. Figure 2c confirms the Raman spectrum, where key features of the wave number are accomplished at about ~368.0 cm^-1^ and 667 cm^-1^ for metal-oxygen (Mn-O and Zn-O) stretching vibrations. These bands can be allocated to a codoped Mn_2_O_3_-ZnO NPs [67].

### Structural properties

**Figure 3:**
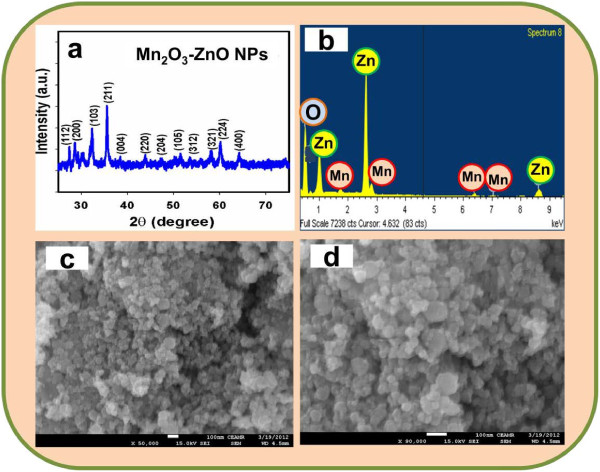
**Investigation of structural, morphological, and elemental analysis for doped Mn2O3-ZnO NPs at room conditions.** (**a**) X-ray powder diffraction (20.0 to 75.0), (**b**) XEDS, and (**c-d**) low to high magnified FESEM images.

Crystallinity and crystal phases of the doped Mn_2_O_3_-ZnO NPs were investigated. X-ray diffraction outlines of codoped NPs are presented in Figure 3a. The Mn_2_O_3_-ZnO NPs samples were examined and exposed as conventional tetragonal structure. The as-grown doped Mn_2_O_3_-ZnO NPs was calcined at 400.0°C in muffle-furnace to start the formation of nano-crystalline phases. Figure 3a shows distinctive crystallinity of the doped Mn_2_O_3_-ZnO NPs and their aggregative crystal arrangement. The reflection peaks in this proto-type were instigated to correspond with Mn_2_O_3_-ZnO NPs phase having tetragonal geometry [JCPDS # 01-024-1133]. The phases employed the key characteristic peaks with indices for calcined crystalline NPs at 2θ values of (112), (200), (103), (211), (004), (220), (204), (105), (312), (321), (224), and (400) degrees. The tetragonal lattice space group is *l41/amd*. Finally, the powder X-ray prototype is corresponded to doped Mn_2_O_3_-ZnO NPs, which may be featured to the lattice site of NPs semiconductor nanomaterials [68-70]. Further, no other impurity peak was found in the XRD prototype screening the codoped Mn_2_O_3_-ZnO NPs phase formation.

The electron dispersive spectroscopy (EDS) evaluation of calcined Mn_2_O_3_-ZnO NPs assigns the existence of Mn, Zn, and O composition in the pure calcined Mn_2_O_3_-ZnO materials. It is clearly employed that NP materials controlled with only manganese, zinc, and oxygen elements, which is shown in Figure 3b. The composition of Mn, Zn, and O is 33.07%, 18.81%, and 48.12% respectively. No other peak related with any impurity has been found in the EDS, which demonstrates that the doped Mn_2_O_3_-ZnO NPs are composed only with Mn, Zn, and O. High resolution FESEM images of calcined Mn_2_O_3_-ZnO NPs are exhibited in Figure 3c-d. The FESEM images displayed of codoped materials with aggregated nano-particles shapes. The average diameter of doped Mn_2_O_3_-ZnO NPs is calculated in the range of 22.7 nm to 50.0 nm, which is close to ~37.5 nm. It is displayed noticeably from the FESEM images that the simple wet-chemical method of prepared crystalline nanomaterials are nanostructure of codoped Mn_2_O_3_-ZnO NPs, which executed in aggregated shape, higher-density, and attained nanostructure in spherical nano-particle shapes. It is also suggested that nanomaterials composed in spherical particle-like morphology of the combined codoped Mn_2_O_3_-ZnO NPs [71,72].

### Chemical analysis

**Figure 4:**
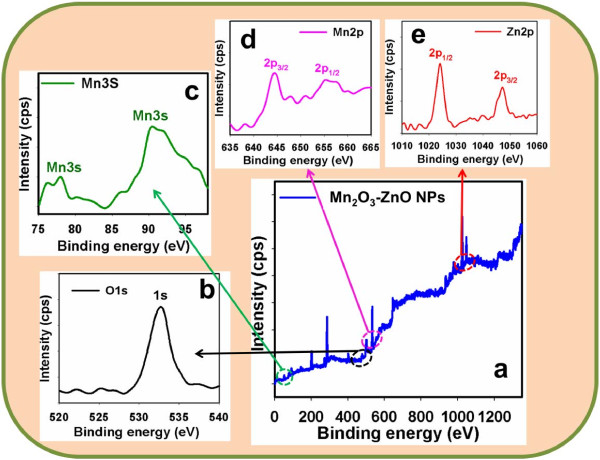
**Evaluation of elemental binding energy (eV) analysis for doped Mn2O3-ZnO NPs with MgKα1 radiation at room conditions.** XPS of (**a**) full-energy scale (0~1350.0 eV), (**b**) O1s level, (**c**) Mn3s level, (**d**) Mn2p level, and (**e**) Zn2p level.

X-ray photoelectron spectroscopy (XPS) is a quantitative spectroscopic method that determines the elemental-composition, empirical-formula, chemical-state and electronic-state of the elements that present in nanomaterials. Here, XPS measurements were employed for doped Mn_2_O_3_-ZnO NPs to examine the chemical states of Zn, Mn, and O atoms. The full XPS spectra of Zn2p, Mn3s, Mn2p, and O1s are displayed in Figure 4a. The O1s spectrum employs a major-peak at 532.9 eV in Figure 4b. The peak at 532.9 eV is assigned to lattice oxygen, may be shown to oxygen (i.e, O_2_^-^) in presence in the doped Mn_2_O_3_-ZnO NP nanomaterials [73]. In Figure 3c, the peaks of the Mn3s binding energy for all the samples displayed at around 77.3 eV and 91.8 eV respectively, which is in excellent conformity with the reference data for Mn [74]. Figure 4d also shows the XPS spectra (spin-orbit doublet peaks) of the Mn2p_(3/2)_ and Mn3p_(1/2)_ regions found with semiconductor doped Mn_2_O_3_-ZnO NPs. The binding energy of the Mn3p_(3*/*2)_ and Mn3p_(1/2)_ peak at 644.7 eV and 655.3 eV respectively indicates the existence of Mn since their bindings energies are similar [75]. In Figure 4e, the spin-orbit peaks of the Zn2p_(1/2)_ and Zn2p_(3/2)_ binding energy for codoped Mn_2_O_3_-ZnO NPs appeared at around 1025.7 eV and 1048.9 eV respectively, which is in good conformity with the reference data for Zn [76].

### Applications: detection of 4-nitrophenol using codoped Mn_2_O_3_-ZnO NPs

**Figure 5:**
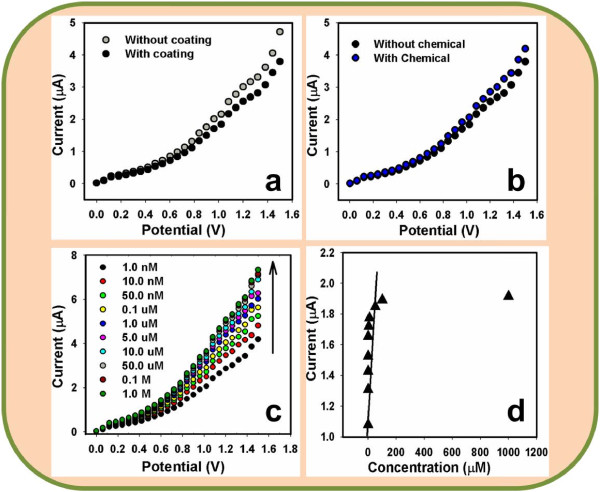
**Detection of 4-nitrophenol using codoped Mn2O3-ZnO NPs by simple and reliable I-V methods.** I-V responses of (**a**) AgE (without coating) and NPs/AgE (with coating); (**b**) NPs/AgE (without chemical) and NPs/AgE (with chemical); (**c**) concentration variation (1.0 nM to 1.0 M) of 4-nitrophenol; and (**d**) calibration plot of NPs/AgE. Potential range: 0 to +1.5 V; Signal-to-Noise Ratio (3N/S) of 3.

Figure 5a exhibits the current responses un-coated (gray-dotted) and coated (dark-dotted) AgE working electrode with doped Mn_2_O_3_-ZnO NPs at room conditions. With doped NPs fabricating surface, the current-signal is reduced compared to without fabricated surface, which reveals the surface is slightly inhibited with doped NPs. The current changes for the codoped Mn_2_O_3_-ZnO NPs modified flat-electrodes before (dark-dotted) and after (blue-dotted) injection of 50.0 μL 4-nitrophenol (~1.0 nM) in 10.0 mL PBS (0.1 M) solution, which is presented in Figure 5b. These considerable changes of surface current are measured in every injection of the target 4-nitrophenol into the electrochemical solution by Keithley electrometer. 10.0 mL of 0.1 M PBS solution is initially transferred into the electrochemical-cell and added the low to high concentration of 4-nitrophenol drop-wise from the stock chemical solution. I-V responses with Mn_2_O_3_-ZnO NPs modified electrode surface were evaluated from the various concentrations (1.0 nM to 1.0 M), which was revealed in Figure 5c. It displays the current changes of developed films as a function of 4-nitrophenol concentration at room conditions. It was also observed that with increasing the concentration of analyte, the resultant currents also enhanced considerably, which corroborated that the response was a surface-process. It shows the response of codoped Mn_2_O_3_-ZnO NPs as a role of analyte concentration at room conditions. A large concentration range of 4-nitrophenol concentration was selected to study the probable investigative parameters, which was calculated in 1.0 nM to 1.0 M. The calibration curve was drawn from the variation of 4-nitrophenol concentrations, which was presented in Figure 5d. It was exhibited a calibration curve for the response current versus 4-nitrophenol concentration of developed doped Mn_2_O_3_-ZnO NPs on AgE electrode. It was measured from the calibration plot that as the concentration of target analyte enhances, the current response also increased and finally at high 4-nitrophenol concentration, the current achieves at a saturated level, which proposes that the active surface sites of doped NPs saturated with analyte units [77]. The sensitivity is manipulated from the calibration-curve, which is close to ~4.6667 μA cm^-2^ μM^-1^. The linear dynamic range of this chemo-sensor exhibits from 0.1 nM to 50.0 μM (linearity, r^2^ = 0.977) and the detection limit was calculated as ~ 0.83 ± 0.2 nM (at an SNR of 3).

Usually, the resistance value of the codoped Mn_2_O_3_-ZnO NPs modified electrodes/chemo-sensors are decreased with enhancing active-surface area, owing to the essential characteristics of the semiconductor materials [78-80]. In fact, oxygen adsorption (O_2_) displays a considerable liability in the electrical features of the doped NPs (n-type semiconductor) structures. Oxygen ion (O_2_^-^) adsorption eradicates the conduction electrons and enhances the resistance of doped Mn_2_O_3_-ZnO NPs. Active oxygen species (i.e., O_2_^−^ and O^−^) are adsorbed onto material surfaces at room condition, and the quantity of such chemo-sorbed oxygen species strongly depend on the structural properties. At room condition, O_2_^−^ is chemo-sorbed, while in NPs morphology, both O_2_^−^ and O^−^ are chemo-sorbed, and the O_2_^−^ vanishes rapidly [81,82]. Here, 4-nitrophenol sensing mechanism of doped Mn_2_O_3_-ZnO NPs chemo-sensor is based on the semiconductors metal oxides, which is held owing to the oxidation/reduction of the semiconductor NPs. According to the dissolved O_2_ in bulk-solution or surface-air of the neighboring atmosphere, the following reactions (vi) & (vii) are accomplished in the reaction medium.

(6) $$e^{-}\left(\mathrm{NPs}\right)+O_{2}\to O_{2}^{-}$$

(7) $$e^{-}\left(\mathrm{NPs}\right)+O_{2}{}^{-}\to 2O^{-}$$

These reactions are consummated in bulk-system/air-liquid interface/neighboring atmosphere owing to the small carrier concentration, which increased the resistance. The 4-nitrophenol sensitivity towards doped Mn_2_O_3_-ZnO NPs could be ascribed to the higher-oxygen lacking conducts to enhance the oxygen adsorption. Larger the amount of oxygen adsorbed on the doped NPs-sensor surface, higher would be the oxidizing potentiality and faster would be the oxidation of 4-nitrophenol. The activity of 4-nitrophenol would have been extremely big as contrast to other toxic chemical with the surface under indistinguishable conditions [83-85]. When 4-nitrophenol reacts with the adsorbed oxygen (by producing electrons) on the chemo-sensor surface, it oxidized to 4-hydroxylaminophenol and water. Later, the oxidation of 4-hydroxylaminophenol takes place to convert to 4-nitrosophenol and the subsequent reversible reduction, which released free electrons (2e^-^) into the conduction-band (C.B.). This phenomenon could be elucidated through the following proposed reactions (viii-x).

(8) $$2O^{-}\to O_{2}+2e^{-}$$

(9) $$\begin{array}{ll} \mathrm{HO} & -\mathrm{Ph}-NO_{2}+4e-+H+\to \mathrm{HO}-\mathrm{Ph}\\ & -\mathrm{NHOH}+H_{2}O \end{array}$$

(10) $$\begin{array}{ll} \mathrm{HO} & -\mathrm{Ph}-\mathrm{NHOH}\to \mathrm{HO}-\mathrm{Ph}-\mathrm{No}\\ & +2_{e}^{-}H^{+}\left(C.B.\right) \end{array}$$

**Figure 6:**
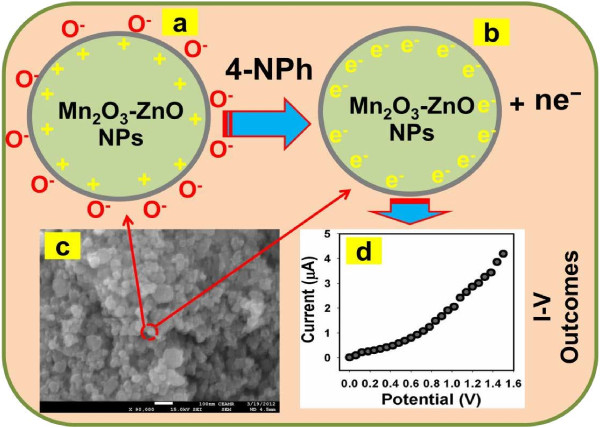
**Mechanism of 4-nitrophenol detection using doped Mn**_**2**_**O**_**3**_**-ZnO NPs coated AgE at room conditions.** (**a**) reduction of NPs/AgE, (**b**) reaction of 4-nitrophenol onto the reduced NPs/AgE, (**c**) FESEM image of NPs, and (**d**) real I-V outcome for 4-nitrophenol detection with NPs/AgE.

These reactions related to oxidation of the reducing carriers in presence of semiconductor doped Mn_2_O_3_-ZnO NPs. These techniques improved the carrier concentration and hence reduced the resistance on exposure to reducing liquids/analytes. At the room condition, the incorporating of metal oxide surface to reduce liquid/analytes results in a surface interceded adsorption process. The abolition of iono-sorbed O_2_ enhances the electron communication and hence the surface conductance of the thin-film [86,87]. The reducing analyte offers electrons co-doped Mn_2_O_3_-ZnO NP surfaces. Accordingly, the resistance is slightly reduced, where conductance is amplified. For this reason, why the analyte response (current) intensified with increasing resultant potential. Thus, the electrons are contributed for quick enhance in conductance of the thin-film. The codoped Mn_2_O_3_-ZnO NPs unusual regions dispersed on the surface would improve the ability of nanomaterial to absorb more O_2_ species giving higher resistances in air ambient, which is presented in Figure 6.

The response time was approximately 10.0 s for the doped Mn_2_O_3_-ZnO NPs coated-electrode to attain saturated-steady state current. The outstanding sensitivity of NPs chemo-sensor can be accredited to good absorption (porous-surfaces coated with conducting binders) and adsorption ability (large-surface area), higher-catalytic activity, and good bio-compatibility of the codoped Mn_2_O_3_-ZnO NPs [88]. Due to large surface area, NPs are proposed a favorable nano-environment for the 4-nitrophenol exposure and gratitude with exceptional sensitivity. The sensitivity of codoped Mn_2_O_3_-ZnO NPs affords high-electron communication characteristics, which improved the direct electron communication between the active sites of NPs and chemo-sensor electrode surfaces [89,90]. The modified thin NPs fabricated-film had a better consistency and reliability. However, owing to large-dynamic surface area, the codoped Mn_2_O_3_-ZnO NPs were entailed productive surroundings for the 4-nitrophenol chemical detection (by adsorption) with huge-amount [91,92]. To check the repeatability and storage stabilities, I-V response for codoped Mn_2_O_3_-ZnO NPs coated chemo-sensor was investigated (up to two weeks). After every experiment, the fabricated chemo-sensor was washed carefully with the PBS buffer solution and executed no considerable reduced on the current responses (recovery, ~95.2%). The sensitivity was retained almost same of initial sensitivity up to week, after that the response of the developed doped Mn_2_O_3_-ZnO NPs sensor gradually decreased. In Table 1, it is contrasted the performances for 4-nitrophenol recognition based doped Mn_2_O_3_-ZnO NPs using various modified electrode materials.

**Table 1:** **Comparison the performances of 4-nitrophenol detection based on doped Mn**_**2**_**O**_**3**_**-ZnO NPs using various reported methods**

**Materials**	**Methods**	**LDR**	**DL**	**Sensitivity**	**Linearity (r**^**2**^**)**	**Ref**
**CuO Nanohybrides**	I-V	1.0 nM to 1.0 mM	0.67 nM.	4.50 μAcm^-2^ mM^-1^	0.7941	[93]
**Poly(safranine) Film Electrode**	CV/LSV	8.0 × 10^−8^ to 4.0 × 10^−5^ M	3.0 × 10^−8^ M	--	0.9880	[94]
**Mn-Doped ZnS QDs**	Chemilumine-scence (CL)	0.1 to 40 μM	76.0 nM	--	--	[95]
**Immunoassay**	Fluorescence Spectroscopy (FL)	5 and 1000 μg/L	3.5 nM	5.7 mg/L	--	[96]
**Graphene Oxide sensors**	CV	0.1 to 120 μM	0.02 μM	--	--	[97]
**B-doped diomond Electrodes**	SWV	--	8.4 mM	0.3943	0.9991	[98]
**Doped Mn**_**2**_**O**_**3**_**-ZnO NPs/AgE**	**I-V method**	**0.1 nM to 50.0 μM**	**~4.6667 μAcm**^**-2**^ **μM**^**-1**^	**~0.83 nM**	**0.9773**	**Current work**

## Conclusions

By reliable I-V techniques for fabricating, assembling and integrating structural semiconductor doped Mn_2_O_3_-ZnO NPs onto conductive flat-silver electrodes has been investigated in details for the detection of toxic 4-nitrophenol compound. Codoped Mn_2_O_3_-ZnO NPs fabricated sensor executed the potential applications in providing 4-nitrophenol chemo-sensors and encouraging improvement has been consummated in this investigation. Besides the development of codoped nanomaterials, there are still a number of significant subjects that are required for additional examination before this nanomaterial can be moved into the profitable uses for the mentioned applications. As for the doped nanostructures, NPs are introduced a route to a new generation of toxic chemo-sensors, but a premeditate effort has to be applied for doped Mn_2_O_3_-ZnO NPs to be taken comprehensively for large-scale applications, and to achieve higher-potential density with accessible to individual chemo-sensors.

## Competing interests

The authors declare that they have no competing interests.

## Authors’ contributions

MMR made a significant contribution to preparation and characterization of doped nanomaterials, and data collection and their analysis and application as well as writing the manuscript. GG has revised the manuscript for intellectual content and corrected accordingly. SAG made a significant contribution to survey the literatures and results. MAD revised the manuscript properly and surveyed the results. SBK participated in the synthesis of samples, collected the data, and contributed in the experimental work. AMA has revised the manuscript for intellectual content. All authors read and approved the final manuscript.
